# Fulminant amoebic colitis: a challenging diagnosis for the surgeon

**DOI:** 10.1093/jscr/rjae724

**Published:** 2024-11-25

**Authors:** David Rafael Barrón-Díaz, Javier Meza-Hernández, Erika Hernández-Montiel, Orlando Solis-Coronado, Jessica Jazmín Betancourt-Ferreyra, Alejandra Núñez-Venzor, Mario Trejo-Ávila, Francisco E Alvarez-Bautista

**Affiliations:** Department of General and Endoscopic Surgery, Dr. Manuel Gea González General Hospital, Calzada de Tlalpan 4800, Belisario Domínguez Sección XVI, Mexico City 14080, Mexico; Department of General and Endoscopic Surgery, Dr. Manuel Gea González General Hospital, Calzada de Tlalpan 4800, Belisario Domínguez Sección XVI, Mexico City 14080, Mexico; Department of General and Endoscopic Surgery, Dr. Manuel Gea González General Hospital, Calzada de Tlalpan 4800, Belisario Domínguez Sección XVI, Mexico City 14080, Mexico; Department of Pathological Anatomy, Dr. Manuel Gea González General Hospital, Calzada de Tlalpan 4800, Belisario Domínguez Sección XVI, Mexico City 14080, Mexico; Department of General and Endoscopic Surgery, Dr. Manuel Gea González General Hospital, Calzada de Tlalpan 4800, Belisario Domínguez Sección XVI, Mexico City 14080, Mexico; Department of General and Endoscopic Surgery, Dr. Manuel Gea González General Hospital, Calzada de Tlalpan 4800, Belisario Domínguez Sección XVI, Mexico City 14080, Mexico; Department of General and Endoscopic Surgery, Dr. Manuel Gea González General Hospital, Calzada de Tlalpan 4800, Belisario Domínguez Sección XVI, Mexico City 14080, Mexico; Department of General and Endoscopic Surgery, Dr. Manuel Gea González General Hospital, Calzada de Tlalpan 4800, Belisario Domínguez Sección XVI, Mexico City 14080, Mexico

**Keywords:** amoebic colitis, amoebic, *Entamoeba histolytica* infection, amoebiasis, fulminant amoebic colitis

## Abstract

Fulminant amoebic colitis is a rare complication of amoebiasis that carries a high mortality rate. Its diagnosis is challenging and requires a high index of suspicion, and its early recognition is a priority to provide timely medical and surgical treatment. We present the case of a male patient who came to the emergency department with unspecific clinical presentation of abdominal pain, systemic inflammatory response and imaging study showing intestinal perforation of the right colon. Fecal peritonitis and perforation at the level of the hepatic flexure were observed, so a right hemicolectomy with terminal ileostomy was performed. Despite adequate medical and surgical treatment, the patient presented progressive deterioration and died. Colon perforation due to *Entamoeba histolytica* was the final diagnosis.

## Introduction

Amoebiasis is a parasitic infection caused by *Entamoeba histolytica (E. hystolytica)*. It is endemic to some tropical regions of the world and is considered the second leading cause of death from parasitic diseases worldwide [1].

The main route of transmission is the ingestion of food contaminated with cysts. Poor hygiene conditions, tropical climates, overcrowding, inadequate fecal waste management, malnutrition, alcoholism, and recent travel to endemic areas are considered risk factors favoring transmission [2].

Most cases are asymptomatic; however, 10%–20% develop amoebic colitis and liver abscesses. Amebiasis can involve any part of the intestine but mainly affects the cecum and ascending colon [3]. Its clinical presentation in case of bowel involvement represents a spectrum, including asymptomatic cases, dysentery cases, and, in extreme cases, fulminant amoebic colitis with peritonitis (FAC) [4, 5].

## Case report

**Figure 1:**
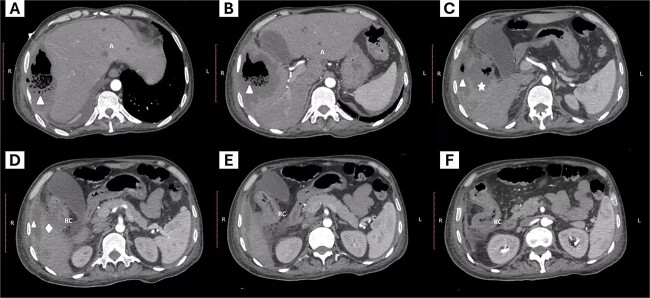
Abdominal computed tomography. The images from left to right show the cephalocaudal sequence of the tomography. (A–C) show a subphrenic abscess (triangle symbol) communicating with a hepatic abscess with compromise of the hepatic parenchyma (star symbol), and the site of colon rupture with communication to the abscess (rhombus symbol). Additionally, peripheral hepatic abscesses are visible (letter A). Images D–E show the site of communication of the colon with the abscessed region and the inflammatory process of the right colon (letters RC).

We present the case of a 44-year-old man with no history of chronic diseases who was admitted to the emergency department with a one-week history of diffuse abdominal pain, predominantly in the right hypochondrium, and fever. Physical examination showed tachycardia (144 beats per minute), blood pressure 120/86 mm/Hg, generalized abdominal pain on palpation without peritoneal signs, and a palpable mass in the right upper quadrant. Laboratory tests showed leukocytosis (17 000/mm^3^), neutrophilia (82%), severe anemia (hemoglobin levels of 6.8 g/dl), platelet count 148 000/mm^3^, serum creatinine 0.7 mg/dl, moderate hyponatremia (128 mmol/L), and hypoalbuminemia (1.6 g/dl). A contrasted abdominal computed tomography was requested as part of his approach, showing thickening of the right colon wall and perforation of the colon wall at the level of the hepatic flexure with communication to a subphrenic abscess with segmental destruction of the right hepatic lobe parenchyma and diffusely distributed hepatic abscesses (Fig. 1).

**Figure 2:**
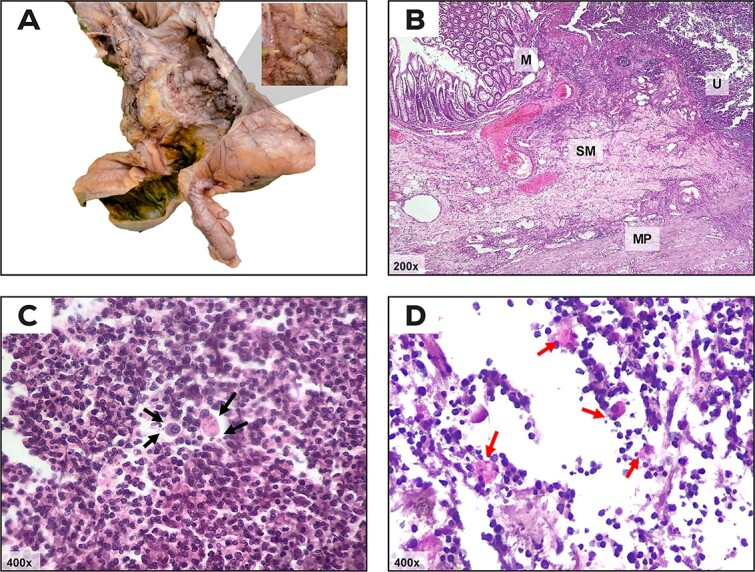
Ulcer associated with amebic colitis by Entamoeba spp. (A) Product of a right hemicolectomy; in the cecum, an ulcer with irregular edges, central depression, and a granular, fibrinous appearance is observed (magnification). (B) Transition from non-lesional colon mucosa to the ulcerated area stained with hematoxylin and eosin (H&E). The ulcer (U) involves mucosa (M) and submucosa (SM) of the colonic wall while sparing the muscularis propria (MP). (C) In the ulcerated area, *Entamoeba* trophozoites are observed, which are ovoid structures with broad, slightly eosinophilic cytoplasm, a small nucleus, and a central intranuclear basophilic dot [karyosome] (arrow symbols), accompanied by lymphocytic-neutrophilic infiltrate (colitis) and cellular debris (H&E staining). (D) Periodic acid-Schiff (PAS) staining; PAS-positive trophozoites with magenta cytoplasm (arrow symbols), some have erythrophagocytosis.

The patient underwent surgery and intraoperative findings included free intestinal fluid in the abdominal cavity, perforation of the colon at the hepatic angle, and a right subphrenic abscess in continuity with the right hepatic lobe. Abdominal cavity lavage and a right hemicolectomy with terminal ileostomy were performed. Histopathological analysis confirmed the diagnosis of acute colitis with infiltration of *E. histolytica* trophozoites (Fig. 2).

As part of the treatment, broad-spectrum antibiotic was started since the admission of the patient, including coverage for gram-negative microorganisms with metronidazole, which is part of the antiamoebic treatment; however, in the postoperative period, the patient developed refractory septic shock that culminated in the patient's death.

## Discussion

Acute amoebic colitis presents with diarrhea, abdominal pain, fever, and blood in the stool. The most feared complication of amoebic dysentery is acute FAC, occurring in 0.5% of cases and carrying mortality of 55% to 88% [6].

Risk factors for developing complicated amoebic infection have been described, including age over 60 years, limited access to potable water, use of corticosteroid therapy, chronic kidney disease and diabetes mellitus [7], concomitant liver abscess, signs of peritonitis, leukocytosis, hyponatremia, hypokalemia and hypoalbuminemia [3]. Our patient lived in an endemic area and had a concomitant liver abscess, leukocytosis, hyponatremia, and hypoalbuminemia as risk factors for the development of this entity.

Serological tests and stool studies can be performed for diagnosis. Cyst visualization can be performed under microscopy, although with low sensitivity (60%). Stool real-time polymerase chain reaction (sensitivity 79% and specificity 96%), stool antigen detection with enzyme-linked immunosorbent assay (ELISA) (sensitivity 55%–100% and specificity 93%–100%) [8, 9], detection of antibodies by serologic assay (indirect fluorescent, counter immunoelectrophoresis or enzyme linked immunosorbent assay) with a sensitivity of 60% to 90% [10] can also be used. No serological tests or stool tests were performed initially on our patient because his clinical presentation required treatment with an emergency surgery, and the diagnosis was confirmed after histopathological analysis of the surgical specimen.

Computed tomography is considered the study of choice in the evaluation of this complication, although the imaging findings tend to be nonspecific as they overlap with those of other conditions, the findings include thickening of the colon wall, intestinal pneumatosis and pneumoperitoneum in case of intestinal perforation [7].

The management of invasive *E. histolytica* infections includes systemic treatment with drugs such as metronidazole, nitazoxanide y secnidazole [8]. Since admission to the emergency department, our patient was started on broad-spectrum antimicrobial therapy with coverage for gran-negative microorganisms, including metronidazole, due to findings in imaging studies suggestive of intestinal perforation.

The diagnosis of this condition is challenging, and histological analysis will have to make differential diagnosis with entities such as intestinal tuberculosis, malignant tumors, and inflammatory bowel disease, being the presence of trophozoites loaded with red blood cells in the thickness of the tissues pathognomonic of an invasive disease [11].

In addition to supportive care and antibiotic therapy, surgical management is a cornerstone in the treatment of FAC, preferring complete resection of the affected segment over conservative surgical treatment due to the risk of suture dehiscence [3].

## Conclusions

Fulminant amoebic colitis is a rare entity whose clinical relevance lies in its high mortality rate related to misdiagnosis. Considering this disease as part of the differential diagnosis in endemic areas and when the patient refers to a history of recent travel to endemic areas increases the diagnostic suspicion, early recognition, and treatment. Surgical management with resection of the affected segment should be considered in case of signs of peritonitis and imaging findings suggesting bowel perforation.

## Conflict of interest statement

The authors declare no conflicts of interest.

## Funding

None declared.
